# Aging anxiety levels of women in menopause and associated factors

**DOI:** 10.1590/1806-9282.20240573

**Published:** 2025-03-17

**Authors:** Sabriye Uçan Yamaç, Nazife Bakir

**Affiliations:** 1 Burdur Mehmet Akif Ersoy University, Bucak School of Health, Department of Midwifery – Burdur, Turkey.; 2 Burdur Mehmet Akif Ersoy University, Bucak School of Health, Department of Nursing – Burdur, Turkey.

**Keywords:** Life, Menopause, Perception, Woman, Old age

## Abstract

**OBJECTIVE:**

The aim of this study was to find out the aging anxiety levels of menopausal women applying to primary healthcare facilities and identify the associated factors.

**METHODS:**

This research was conducted on a face-to-face basis with 527 menopausal women applying to family health centers in the district of Serik in Antalya province of Turkey from March 1, 2022, to July 1, 2022, and agreeing to participate in the study. After participant women's sociodemographic and menopausal data were recorded, the Aging Anxiety Scale for Menopausal Women was administered to all participant women.

**RESULTS:**

Of 527 participant women, 41.7% were aged 50–57 years, 71.7% were elementary school graduates, 76.5% were married, 63.0% were housewives, 54.6% were from nuclear families, 49.3% spent a large part (two-thirds) of their lives in the district, 52.8% had incomes equaling their expenses, 69.3% were living with their spouses and children, and 35.7% had their last period 4 years ago or earlier. The mean scores obtained by women from the Aging Anxiety Scale for Menopausal Women and its Fear of Old People, Fear of Loss, Physical Appearance, and Psychological Concerns sub-scales were successively 30.78 (8.00), 10.55 (5.75), 6.75 (3.56), 5.81 (3.42), and 7.63 (2.96).

**CONCLUSION:**

In the identification and management of areas of top priority for the delivery of health services, a significant role falls on midwives and nurses, who are supposed to approach an individual with a biopsychosocial perspective.

## INTRODUCTION

Menopause is an inevitable developmental event encountered by women aged 42–54 years. The fall in estrogen levels, together with the end of menstrual cycles, is associated with multiple vasomotor, physical, neuropsychological, and sexual symptoms that are likely to negatively affect the quality of life^1,2^. A variety of symptoms that are likely to negatively affect the quality of life depending on aging and hormonal changes can be observed in the majority of women in menopause^3^. The most common symptoms are vasomotor symptoms such as hot flashes and night sweats, musculoskeletal system problems, sleep disorders, mood changes, irritable bladder, atrophy in breasts and reproductive organs, sexual dysfunctions such as dyspareunia and decreased libido, and cardiovascular system diseases^4,5^. The number and severity of these symptoms change depending on the woman's menopause age, the way in which the woman goes through menopause, the woman's knowledge and attitude, the culture where the woman lives, and the woman's gender roles^6^. Menopausal symptoms can even lead to severe mental illnesses in women, as well as decreasing the quality of life^7,8^. Menopause, which gives rise to aging anxiety in women, is an attention-catching condition that is defined as having no period for a minimum of 12 months^9^. The process that starts before menopause and continues for a few years is called perimenopause^10^. In the perimenopause period, hormonal changes can lead to irregular menstrual cycles, an increase in fat mass and body weight, a decrease in tissue elasticity, an increase in wrinkles, changes in skin tone and tissue, and differences in hair color and thickness. Such sort of changes during the perimenopause period can induce women to feel worried about aging, dissatisfied with their bodies, and ashamed of their bodies^9,11^. The postmenopause period refers to the period after the end of reproductive function^9^. In the postmenopause period, certain women perceive the loss of reproductive ability as aging^12^. In the study by Araya et al., women perceived menopause as the end of the reproductive process and the transition to old age^13^. So that women going through menopause can consider aging positively and adapt to this situation, it is obligatory to address aging anxiety appropriately. Therefore, this study aimed to identify the aging anxiety levels of women in menopause and the associated factors.

## METHODS

### Research model

The research is descriptive and cross-sectional.

### Population and sample

The research population is all women who were aged 42–64 years, were in the premenopausal or postmenopausal period, and applied to family health centers in the district of Serik in Antalya province of Turkey from March 1, 2022, to July 1, 2022. Before the research, by taking the effect size (f) as 0.20, alpha level (α) as 0.05, and power (1–beta) as 0.95, the minimum sample size for the research was calculated as 470 with G*power 3.1.9.7, which is a tool for statistical power analysis. Considering the likely data losses during the research process, research data were collected from 540 women. As certain women provided inaccurate or missing data, the research was completed with 527 women.

### Data collection tools

#### Personal information form

The Personal Information Form was created by the researchers based on the literature^6,14–16^ and contains data on the descriptive characteristics of the participant women.

#### The Aging Anxiety Scale for Menopausal Women

Lasher and Faulkender^17^ developed this scale originally as the Anxiety About Aging Scale (AAS) to measure aging anxiety levels. It has 20 items and 4 sub-scales (Fear of Old People, Fear of Loss, Physical Appearance, and Psychological Concerns). Kalaycı^14^ adapted the AAS to Turkish to use it on women in menopause and titled it the Aging Anxiety Scale for Menopausal Women (AASMW). It is a 5-point Likert-type scale (Strongly Agree, Agree, Neither Agree Nor Disagree, Disagree, and Strongly Disagree). The Turkish form of the scale (AASMW) consists of 16 questions (Fear of Old People Sub-Scale: Items 1, 2, 6, 9, and 15; Fear of Loss Sub-Scale: Items 3, 5, 10, and 13; Physical Appearance Sub-Scale: Items 8, 11, and 16; Psychological Concerns: Items 4, 7, 12, and 14), and the minimum and maximum total scores to be obtained by a respondent from the AASMW are successively 16 and 80 points. A high total AASMW score shows that the respondent has high-level aging anxiety. Cronbach's alpha coefficient was found to be 0.828 for the Turkish form of the scale. In this research, Cronbach's alpha coefficient was identified as 0.75 for the AASMW.

### Data collection

For the research, first of all, the ethical endorsement was obtained from the Non-Invasive Clinical Research Ethics Committee of the Burdur Mehmet Akif Ersoy University of Turkey, and permission was also received from the institution where the research would be conducted. The research data were collected from March 1, 2022, to July 1, 2022, by using the face-to-face interview method. The survey form had three parts. In the first part of the survey form, after a participant woman was informed about the research, she expressed her consent to participate in the study through an informed consent form. The second part contained the Personal Information Form developed by researchers, while the third part presented the AASMW. It took each participant woman approximately 10–15 min to fill out the survey form.

### Data analysis

The Statistical Package for Social Science 25.0 was used for the data analysis. The Kolmogorov-Smirnov test was utilized to determine whether the research data were normally distributed. The one-way analysis of variance was used in the comparison of more than two groups, and Bonferroni correction was utilized as a post-hoc test to find which group(s) had a statistically significant difference from other groups. The independent sample t-test was used in the comparison of two groups. In the research, 0.05 was set as the cut-off point for statistical significance (p<0.05).

## RESULTS

Table 1 shows the breakdown of women's descriptive characteristics. In this respect, of all participant women, 41.7% were aged 50–57 years, 71.7% were elementary school graduates, 76.5% were married, 63.0% were housewives, 54.6% were from nuclear families, 49.3% spent a large part (two-thirds) of their lives in the district, 52.8% had incomes equaling their expenses, 69.3% were living with their spouses and children, and 35.7% had their last period 4 years ago or earlier.

**Table 1:** The breakdown of women's descriptive characteristics.

Descriptive characteristics		Number (percentage)
Age		
	42–49 years	172 (32.6)
	50–57 years	220 (41.7)
	58–64 years	135 (25.6)
Education status		
	Elementary school	378 (71.7)
	Middle school	87 (16.5)
	High school	50 (9.5)
	University	12 (2.3)
Marital status		
	Married	403 (76.5)
	Widow/divorced	84 (15.9)
	Single	40 (7.6)
Employment status		
	Housewife	332 (63.0)
	Working	195 (37.0)
Family type		
	Nuclear family	288 (54.6)
	Extended family	239 (45.4)
The place where the woman spent a large part (two-thirds) of her lifespan		
	Village/town	80 (15.2)
	District	260 (49.3)
	Province center	187 (35.5)
Income status		
	Income below expenses	151 (28.7)
	Income equaling expenses	278 (52.8)
	Income above expenses	98 (18.6)
With whom the woman lives		
	Spouse	24 (4.6)
	Child	77 (14.6)
	Spouse and child	365 (69.3)
	Alone	50 (9.5)
	Others	11 (2.1)
Date of the last period		
	1 week–15 days ago	151 (28.7)
	1–3 months ago	35 (6.6)
	4–11 months ago	18 (3.4)
	1–3 years ago	136 (25.8)
	4 years ago or earlier	187 (35.7)
Total		527 (100)

The mean scores obtained by women from the AASMW and its Fear of Old People, Fear of Loss, Physical Appearance, and Psychological Concerns sub-scales were successively 30.78 (8.00), 10.55 (5.75), 6.75 (3.56), 5.81 (3.42), and 7.63 (2.96) (Table 2).

**Table 2:** The mean scores obtained by women from the Aging Anxiety Scale for Menopausal Women and its sub-scales.

	Mean	SD	Minimum	Maximum
Fear of Old People Sub-Scale	10.55	5.75	5.00	22.00
Fear of Loss Sub-Scale	6.78	3.56	4.00	18.00
Physical Appearance Sub-Scale	5.81	3.42	3.00	15.00
Psychological Concerns Sub-Scale	7.63	2.96	4.00	18.00
AASMW	30.78	8.00	16.00	70.00

Table 3 indicates the breakdown of women's mean AASMW scores by their descriptive characteristics. In this regard, it was found that women aged 58–64 years obtained a lower mean AASMW score than women in other age groups, while women who were middle school graduates obtained a higher mean AASMW score than women at other levels of education, and these differences were statistically significant. Also, women who were single, women who were working, and women who were from nuclear families obtained significantly higher mean AASMW scores than the other respective groups of women. Moreover, women who had incomes equal to their expenses obtained a significantly higher mean AASMW score than women at other income levels. Besides, women who were living with their spouses and women who were living with other individuals obtained significantly higher mean AASMW scores than the other respective groups of women. Women who had their last period 1–3 months ago obtained a significantly higher mean AASMW score than women who had their last period 1 week to 15 days ago and women who had their last period 4–11 months ago. Likewise, women who had their last period 4 years ago or earlier obtained a significantly higher mean AASMW score than women who had their last period 1 week to 15 days ago (p=0.000).

**Table 3:** The breakdown of women's mean Aging Anxiety Scale for Menopausal Women scores by their descriptive characteristics.

Descriptive characteristics		Mean (SD)	F/t	Statistically significant difference/p
Age*	42–49 years a	31.72 (9.94)	F: 8.731	a,b>c
	50–57 years b	31.55 (7.13)		**p: 0.000**
	58–64 years c	28.34 (5.84)		
Education status*	Elementary school a	29.57 (6.65)	F: 20.954	b>a,c
	Middle school b	36.65 (7.67)		**p: 0. 000**
	High school c	29.62 (11.81)		
	University d	31.16 (11.76)		
Marital status*	Married a	30.91 (7.83)	F: 16.034	c>a>b
	Widow/Divorced b	27.64 (7.21)		**p: 0.000**
	Single c	36.05 (8.46)		
Employment status**	Housewife	30.21 (8.03)	t:−2.157	**p: 0.031**
	Working	31.76 (7.89)		
Family type**	Nuclear family	31.83 (9.51)	t: 3.329	**p: 0.001**
	Extended family	29.52 (5.44)		
The place where the woman spent a large part (two-thirds) of her lifespan*	Village/town a	30.88 (7.87)		
	District b	30.75 (7.60)	F: 0.008	p: 0.912
	Province center c	30.78 (8.62)		
Income status*	Income below expenses a	29.73 (9.55)	F: 9.285	b>a,c
	Income equaling expenses b	32.13 (7.92)		**p: 0.000**
	Income above expenses c	28.57 (3.94)		
With whom the woman currently lives*	Spouse a	43.37 (11.52)	F: 22.397	a,e>b,c,d
	Child b	30.14 (8.27)		
	Spouse and child c	29.99 (7.09)		**p: 0.000**
	Alone d	29.62 (6.64)		
	Others e	39.36 (1.91)		
Date of the last period*	1 week–15 days ago a	28.35 (8.41)	F: 7.929	b>a,c: e>a
	1–3 months ago b	34.94 (7.58)		**p: 0.000**
	4–11 months ago c	28.50 (13.33)		
	1–3 years ago d	30.87 (4.04)		
	4 years ago or earlier e	32.12 (8.67)		

SD: Standard deviation,

* One-way ANOVA post-hoc test: Bonferroni correction,

** Independent samples t-test. Bold indicates significant difference p<0.05.

## DISCUSSION

As it is estimated that the number of women in menopause will reach 1.2 billion across the world by 2030, it is of critical importance to understand how menopause will affect a woman's emotional health and how many women are affected by menopause^15,18^. Just as aging anxiety is a significant mediating factor in attitudes and behaviors toward older individuals, it is also a mediating factor in a person's adaptation to his/her own aging processes^19^. In the relevant literature, there are studies finding that the prevalence of anxiety disorder was high among women in menopause and that its severity was associated with the severity of menopause syndrome^20,21^. Also, in the relevant literature, it was found that stress, anxiety, and depression levels of women in menopause increased as their menopause-related complaints and social appearance concerns increased^16^. In our study, the mean of women's AASMW scores was 30.78 points, and hence, women had medium-level menopause-related aging anxiety. In this context, the results of our study are similar to those obtained in the relevant literature. Identifying aging anxiety in women going through menopause is important, as it will guide these women toward psychological, social, and health-related support mechanisms in the early period^14^. In our study, it was discerned that the fear of aging-related loss, anxiety about physical appearance, and aging-related psychological concerns were lower.

Depressive and anxiety symptoms are more frequently observed in the menopausal transition and postmenopausal periods than they are observed in the reproductive period. Depressive symptoms are more common than anxiety symptoms. Screening and evaluating depressive and anxiety symptoms in perimenopausal women are important, particularly for women with high-risk factors^2^. Menopause is the beginning of aging in women. In this process, certain women experience physical changes that are likely to affect their psychological and social conditions, as well as the quality of their lives^22^. In our study, it was found that women who were single, women who were working, and women who were from nuclear families obtained higher mean AASMW scores. Single women may have had high levels of aging anxiety as they experienced the fear of aging or had anxiety about aging alone before the fulfillment of a life goal such as getting married or having a child.

Health workers are expected to treat not only physical problems but also psychological problems in the context of health services directed to women, particularly during the premenopause period.

## CONCLUSION

The results of various studies conducted until today uphold that women in menopause feel aging anxiety and worry. In our study, it was discerned that there were women who had medium-level anxiety and worry. The results of our study show that women should be given information on the likely problems and coping methods to ensure that they can have more comfortable, healthy, and peaceful premenopause and postmenopause periods. It is quite important that, particularly, nurses and midwives adopt a holistic approach toward women going through menopause.
